# Thymic function and T cell parameters in a natural human experimental model of seasonal infectious diseases and nutritional burden

**DOI:** 10.1186/1423-0127-18-41

**Published:** 2011-06-15

**Authors:** Pa T Ngom, Juan Solon, Sophie E Moore, Gareth Morgan, Andrew M Prentice, Richard Aspinall

**Affiliations:** 1 Nutrition Programme, MRC Laboratories, The Gambia; 2 MRC International Nutrition Group, London School of Hygiene and Tropical Medicine, Keppel Street, London WC1E 7HT, UK; 3 Translational Medicine, Cranfield Health, Cranfield, UK

## Abstract

**Background:**

The study exploits a natural human experimental model of subsistence farmers experiencing chronic and seasonally modified food shortages and infectious burden. Two seasons existed, one of increased deprivation and infections (Jul-Dec), another of abundance and low infections (Jan-Jun); referred to as the hungry/high infection and harvest/low infection seasons respectively. Prior analysis showed a 10-fold excess in infectious disease associated mortality in young adults born in the hungry/high infection versus harvest/low infection season, and reduced thymic output and T cell counts in infancy. Here we report findings on the role of early life stressors as contributors to the onset of T cell immunological defects in later life.

**Methods:**

We hypothesised that season of birth effects on thymic function and T cell immunity would be detectable in young adults since Kaplan-Meier survival curves indicated this to be the time of greatest mortality divergence. T cell subset analyses by flow-cytometry, sjTRECs, TCRVβ repertoire and telomere length by PCR, were performed on samples from 60 males (18-23 y) selected to represent births in the hungry/high infection and harvest/low infection

**Results:**

Total lymphocyte counts were normal and did not differ by birth season. CD3^+ ^and CD4^+ ^but not CD8^+ ^counts were lower for those born during the hungry/high infection season. CD8^+ ^telomere length also tended to be shorter. Overall, CD8^+ ^TCRVβ repertoire skewing was observed with 'public' expressions and deletions seen in TCRVβ12/22 and TCRVβ24, respectively but no apparent effect of birth season.

**Conclusions:**

We conclude that, although thymic function was unchanged, the CD4^+ ^and CD3^+ ^counts, and CD8^+ ^telomere length results suggested that aspects of adult T cell immunity were under the influence of early life stressors. The endemicity of CMV and HBV suggested that chronic infections may modulate immunity through T cell repertoire development. The overall implications being that, this population is at an elevated risk of premature immunosenescence possibly driven by a combination of nutritional and infectious burden.

## Background

A large retrospective community-based study using demographic data generated over a 50 year period from 3102 individuals born in alternating seasons of relative food availability and low infectious diseases burden (harvest/low infection; January to June) and deprivation and high infectious diseases (hungry/high infection season; July to December), showed that those born in the hungry/high infection were 10-times more likely to die from infectious diseases as young adults[1,2]. By splitting the year in half, seasonal fluctuations are taken into account, ensuring that periods of typical hungry/high infection and harvest/low infection, were clearly included in the right category. In the absence of overt droughts which are rare in The Gambia, this categorization is considered sufficient safeguard for possible year to year variations of the seasons. Follow up studies revealed an association between enhanced thymic function and being born in the harvest/low infection season for 8 week old infants[3]. This suggests that seasonal variation in nutrition supplies and infectious diseases may modulate immunity through the thymus from early in life; potentially persisting to adolescence and accounting for the reported season of birth differences in mortality rates[2].

In experimental animals, the detrimental effects of malnutrition and infection on immunity have long been recognised[4-6]. In 2-59 month old children, *plasmodium falciparum *specific IgG antibody responses are compromised in the malnourished[7]. Single micronutrient deficiency, for example of zinc, has been associated with poor pneumonia outcome, improved by zinc supplementation[8]. Selenium deficiency is associated with myocardial infarction caused by coxsackie B virus which is inhibited by selenium;[9] and selenium supplementation also reverses the symptoms of AIDS,[10] in which selenium deficiency is common[11,12]. Vitamin D deficiency also spells poor innate immunity through modulation of neutrophil and macrophage function; and vitamin D status is associated with respiratory illness and risk of TB[13,14]. Thymic atrophy characterises diet induced malnutrition in mice;[15] and the administration of the satiety hormone, leptin which acts via the nutritional-status-sensitive[16] hypothalamic-pituitary-adrenal axis, has been shown to reverse starvation induced thymic involution[15]. The thymus is also a target for disease causing pathogens, and the exposure of mice to *plasmodium berghei*, resulted in invasion of the thymus by day 14; accompanied by severe thymic atrophy[17].

In humans, postmortem studies show thymic involution in the severely malnourished[18]. Furthermore, cytokines including IL-7 and IL-2 which are important for thymic and T cell development may be suppressed by changes in the thymus[19,20]. In children, reduced CD4^+^CD62L^- ^and CD8^+^CD28^-^effector T cells in the healthy as well as the malnourished-infected, compared to the well-nourished-infected are seen[21]. The human thymus is also vulnerable to infections, and thymic size was significantly decreased in children infected with HIV[22]. Reports show that a smaller thymus was a consistent and independent risk factor for mortality and was predictive of immune competence[23,24].

Our original analysis of mortality by season of birth revealed the surprising observation that the Kaplan-Meier survival curves only started to diverge in adolescence[2] suggesting that any initial deficits in immunological endowment are magnified by an accelerated immunosenescence and only fall below the protective threshold in early adulthood. To test this possibility we recruited two groups of young adults (18-23 y) born in the hungry/high infection and harvest/low infection and, based on the known susceptibility of the thymus to nutritional insult and our previous evidence for early-life effects[3]. We investigated T cell numbers, sjTRECs, T-cell repertoire and telomere lengths. We assumed chronic and seasonal nutritional deprivation existed, partly because of the low dietary intake and the hungry/high infection and harvest/low infection seasonal cycles of weight lost and gain observed for the past decades[25]. Growth was also reported to deteriorate in infants during the hungry/high infection [26] accompanied by serious depletion of staple foods. Infections including malaria and diarrhea are endemic here, with the highest prevalence in the hungry/high infection season[27-29].

The current study of young adults exposed both at birth and repeatedly for the years leading to adolescence, presents a natural human experimental model which could be exploited for the characterisation of the immunological mechanisms underlying the effects of seasonal fluctuations as well as chronic, nutritional deprivation and infectious burden. Subsistence cultivation of crops for food, practised in this community, is consistent with a chronic lack of adequate nutrition. Furthermore, farming here is limited to the annual rains. Consequently staple food supplies are depleted for much of the year as the produce of the farming season is exhausted before the next crop matures; this occurs amidst heavy manual labour from the early teenage years, probably worsening overall nutritional/energy status, with environmental conditions conducive to the propagation of infections. We predict that the exposure of both the mothers and their fetuses during pregnancy, and of their babies after birth, to deprivation and infectious burden may have a synergistic effect on the maturing immune system and long term health of those born during the hungry/high infection season. Therefore the overall effect is that residents are under both a general and chronic (brought about by the limitations of subsistence farming and the repeated annual cycles, endured from early life through to adolescence), as well as a seasonally differential risk of nutritional deprivation and infectious burden. We report suggestions that aspects of adult T cell immunity may be under the influence of early life stressors.

## Methods

A prospective cohort study of 60 overtly healthy 18-23 year (average age 21.3 y, SD 2.0 y) old men living in rural village community clusters, born in the hungry/high infection (n = 30; average age 21.1 y, SD 1.9 y) or harvest/low infection (n = 30; average age 21.5 y, SD 2.2 y) season, was conducted. Thirty milliliters venous blood was taken following signed informed consent from each participant. Ethical approval was granted by the joint MRC and Gambian Government Ethics Committee (Reference number SCC 863).

### Lymphocyte subset analysis

Lymphocyte subsets were evaluated by flowcytometry using the FACsCalibur [Becton Dickinson UK Ltd, Oxford, UK] following monoclonal antibody staining. Briefly, 100 μl whole blood was incubated with 10 l monoclonal antibodies including anti-CD4^+^, CD8^+ ^or CD3^+ ^[Cyto-stat, Beckman Coulter S.A, Nyon Switzerland]. The red blood cells were lysed and the white blood cells fixed and stabilized [Q-prep Beckman, Coulter] then stored at +4°C prior to transportation on ice to the base laboratory for analysis.

#### CD4^+ ^and CD8^+ ^cell selection and Triazol treatment

PBMCs were separated by ficoll gradient centrifugation followed by positive selection of CD4^+ ^and CD8^+ ^T cells using magnetic beads [MACS columns, Miltenyi Biotec], then spun at 2000 rpm for 5 minutes. The pellet was re-suspended in 1 ml Triazol reagent (SIGMA), then store at -80°C until use.

#### DNA/RNA extraction and cDNA generation

The Triazol treated samples were thawed and 1 ml mixed with 0.2 ml chloroform followed by centrifugation at 14,000 rpm for 15 minutes to separate into an aqueous RNA phase, an organic protein layer and a DNA interphase.

RNA was extracted by adding 0.5 ml isopropanol to the aqueous phase and incubating at-20°C overnight, then centrifuged at 14000 rpm for 10 minutes. The resulting RNA pellet was washed in 1 ml of 75% ethanol, dried on ice for 5-10 minutes then rehydrated in 10 μl sterile water. cDNA was generated by RT PCR using oligo dT primers.

DNA was extracted by mixing the inter phase with 0.3 ml of 100% ethanol, then centrifuged at 9000 rpm for 10 minutes and the pellet washed twice in 1 ml of 0.1 M sodium citrate containing 10% ethanol; followed by 1 ml of 70% ethanol. The DNA pellet was dried and rehydrated in 100 μl sterile water, then DNA concentration determined by spectrophotometry.

Signal joint (sj) T cell receptor (TCR) rearrangement excision circles (TREC) analysis The sjTREC assay has been previously described in detail[3]. Briefly: 2 μl of DNA from standards and samples were added to 18 μl of master mix containing 0.3 μM of sjTREC specific forward: GCCACATCCCTTTCAACCATGCTGAC and reverse: TTGCTCCGTGGTCTGTGCTGGCATC primers, 5 mM MgCl_2_, 200 ng/μl BSA or 0.01% Tween 20 to give a total reaction volume of 20 μl. The reactions were then transferred into glass capillary tubes for real time PCR quantification of sjTRECs, using the Light Cycler. The conditions for the real time PCR were as follows: 1 cycle of 95°C for 15 minutes for Taq polymerase activation, followed by 40-60 cycles of 95°C for 1 second; annealing at 62°C for 25 seconds; amplification at 72°C for 12 seconds and measurement of fluorescence emitted from product at 85°C for 5 seconds. A cloned sjTREC fragment of known concentration was used as standard and could also serve as a positive control. Sterile distilled water was included in each reaction to serve as a negative control.

### Expressed TCRVβ repertoire

The lack of abnormal clonal expansion for the CD4^+ ^TCRVβ repertoire reported by others, [[30], and [31]] prompted us to restrict the repertoire analysis to the CD8^+ ^TCRVβ. Following RA extraction and reverse-transcription to cDNA, products of the first round PCR generated using the 24 TCRVβ and TCRCβ primers (24 reactions per T cell subset per subject) were confirmed on agarose gel to be of the expected CDR3 lengths, ranging from 100 bp to 400 bp[32]. Following this confirmation and labelling of DNA products with fluorescent sequencing dye, the CDR3 length distribution of the T cell clones within each of the 24 TCRVβ types were determined by spectra typing using a gene scanning approach[32]. T cell TCRVβ repertoire assay is described in detail elsewhere[32]. Briefly, 24 TCRVβ and one TCRCβ specific primers were used to amplify cDNA corresponding to amino acid residues 95-106 of the TCRVβ CDR3 region. The product was labeled with a 5'FAM dye conjugated TCRCβ specific primer. The product was scanned using an ABI PRISM ^® ^310 sequencer (GMI, Inc USA); to generate a spectra type of peaks representing the different T cell clones in each sample.

### Telomere length estimation

The telomere length assay is based on the method by Cawthon et al[33]. Briefly, commercially obtained telomere specific primers; CGGTTTGTTTGGGTTTGGGTTTGGGTTTGGGTTTGGGTT (forward) and GGCTTGCCTTACCCTTACCCTTACCCTTACCCTTACCCT (reverse), were used to amplify telomeric DNA in the CD4^+ ^and CD8^+ ^T cell subset. Six serial dilutions of standards containing telomeric DNA of known concentration were prepared by doubling dilution. Sample DNA and standard were then placed in 0.2 μl tubes and heated at 95°C for 5 minutes, and then snap chilled on ice for ≥5 minutes. Real time PCR reactions were set up as follows: A master mix made by adding 10 μl of 2 × QuantiTect mix [Qiagen, UK], 250 nM each of the telomere primer pairs 1% DMSO for increased primer binding specificity and 2.5 mM DTT for increased Taq DNA polymerase enzyme fidelity. Then 2 μl of sample DNA containing 35 ng, was added to 18μl master mix then transferred to glass capillaries for the real time PCR analyses. Optimal PCR conditions were achieved at 1 cycle of 95°C for 15 minutes initial denaturation, followed by 35 cycles of 95°C for 15 seconds denaturation; 54°C for 40 seconds simultaneous primer annealing and extension followed by 1 cycle of 65°C for 5 seconds fluorescence measurement. Results were generated as cross over time (C_t_)/CD4^+ ^or CD8^+ ^T cell, where C_t _was the time in seconds needed to generate sufficient telomere DNA product for detection by the Light Cycler [Rouche diagnostics, UK]. The smaller the C_t _the more telomere repeat sequences, hence the longer the telomere in the starting DNA sample.

### Statistical analysis

For the T cell repertoire analysis, the Kolmogorov Smirnov test was used to assess variation in the distribution of T cell clones within the population. For the season of birth analyses, means were compared for those born in the hungry/high infection season versus those born in the harvest/low infection season. For normally distributed data, the Student's t test was used, and for skewed data, log transformation was applied and the Man Whitney U test used and geometric means (GM) given. P < 0.05 was considered statistically significant.

## Results

The mean birth weight of the population was 3.07 Kg, ranging from 1.64-3.65 Kg. There were only 2 subjects with low birth weight (<2.5 kg). To evaluate the effects of high or low birth weight as indicators of nutritional status, we categorized by birth weight above (high) or below (low) the population median, and subjected the data to analyses of co-variance; but there were no overall differences in birth weight effects (data not shown).

Thymic output and T cell subsets in the population

The thymic output, repertoire and telomere length analyses were based on CD4^+ ^and CD8^+ ^sorted cells with purity of at least 90%. Of the 60 subjects included, 56 (27 hungry/high infection and 29 harvest/low infection) CD4^+ ^T cell and 59 (29 hungry/high infection and 30 harvest/low infection) CD8^+ ^T cell samples had complete sjTREC data. Samples from 4 of the CD4^+ ^and 1 from the CD8^+ ^subset could not be analysed for sjTRECs due to poor sample quality and therefore were excluded.

As a molecular marker of thymic output, sjTRECs concentrations in peripheral blood samples were used to evaluate thymic function. Mean sjTREC level for the CD4^+ ^T cell subset was approximately 1.5 fold higher than for the CD8^+ ^T cell subset, reflecting a CD4^+^:CD8^+ ^production ratio of approximately 1.5 Table 1). However, the difference was not statistically significant. The results also showed that sjTREC levels of neither the CD4^+ ^nor the CD8^+ ^T cell subsets differed significantly by season of birth (Table 1).

**Table 1:** sjTREC levels in the population and by season of birth 18-23 year old men.

		GM sjTREC/100 T cells			
	**CD4^+ ^** **(n)**	**95% CI**	**CD8^+ ^** **(n)**	**95% CI**	*P value

**All**	0.061 (56)	0.03-0.11	0.043 (59)	0.02-0.8	0.44

**Harvest**	0.060 (29)	0.03-0.14	0.029 (30)	0.01-0.07	0.27
**Hungry**	0.063 (27)	0.03-0.15	0.064 (29)	0.03-0.15	0.96
**P value**	0.95		0.18		

sjTREC/100 CD4^+ ^and CD8^+ ^T cells of positively selected PBMCs are shown for the whole population. The Man Whitney U test was used with geometric means (GM) compared for the two seasons of birth. The number of samples (n), 95% CI and p values are also shown. sjTREC data were considered unreliable for the CD4^+ ^and CD8^+ ^samples of 4/60 and 1/60 subjects respectively, therefore these were rejected.

Since thymic output influences peripheral T cell numbers, the major peripheral T cell subsets were similarly analyzed. All but 2 subjects had complete lymphocyte count data (29 from each season); while 59 (30 hungry/high infection and 29 harvest/low infection) had full CD4^+ ^and CD8^+ ^counts. There were 57 (29 hungry/high infection and 28 harvest/low infection) subjects with complete CD3^+ ^counts. There were no significant differences in the percentage CD4^+^, CD8^+ ^or CD3^+ ^T cell subsets by season of birth. However, the absolute numbers of CD4^+ ^and CD3^+ ^T cells were significantly lower in those born during the hungry/high infection compared to the harvest/low infection season (Table 2). The CD4^+^:CD8^+ ^ratio was similar for both harvest/low infection and hungry/high infection season born, at 1.6 and 1.5 respectively.

**Table 2:** Lymphocyte phenotypes, in the population by season of birth in 18-23 years old men.

	Lymphocyte subsets (SD) [95%CI]							
	Mean Lymph %	GM Lymph × 10^6^/μl	Mean CD4^+ ^ %	GM CD4^+ ^ × 10^6^/μl	Mean CD8^+ ^ %	GM CD8^+ ^ × 10^6^/μl	Mean CD3^+ ^ %	GM CD3^+ ^ 10^6^/μl
**All**	41.7	2.8	40.4	1.0	25.7	0.6	74.2	1.8
	(9.1)	[2.6-2.9]	(7.4)	[1.0-1.1]	(6.4)	[0.6-0.7]	(8.0)	[1.8-1.9]
**n**	58	58	59	59	58	59	57	57

**Harvest**	41.9	2.8	41	1.3	25.1	0.7	74.6	2.1
	(7.6)	[2.6-3.1]	(7.2)	[1.0-1.3]	(6.2)	[0.6-0.8]	(8.7)	[1.9-2.3]
**n**	29	29	29	29	28	29	28	28
**Hungry**	42.9	2.56	39.9	1.0	26.4	0.6	73.8	1.8
	(9.2)	[2.3-2.7]	(7.6)	[0.9-1.1]	(6.7)	[0.6-0.8]	(7.4)	[1.6-2.0]
**n**	29	29	30	30	30	30	29	29
**p value**	0.63	0.06	0.57	<0.03	0.44	0.43	0.70	<0.05

Means and GM in the whole population and by season are shown. The number of samples (n), 95% CI and p values comparing the two seasons are also shown.

### CD8^+ ^TCRVβ size distribution showed extensive repertoire distortion with season of birth effects on TCRVβ 12/24

CD8^+ ^TCRVβ repertoire data was available for 52 subjects, with data from 4 subjects missing from each group. While the CD8^+ ^T cell subset exhibited extensive distortions (visually) in the spectra types of most of the 24 TCRVβ types analyzed, it emerged from the initial analyses that the CD4^+ ^T cell subset consistently exhibited normal spectra type distributions consequently further repertoire analysis was limited to the CD8^+ ^T cell subset. Although the overall variability in the distribution of T cell clones assessed by the Kolmogorov Smirnoff test, which measures divergence of the distribution from the expected normal, did not show significant season of birth differences (p < 0.67), effects on individual TCRVβ types were observed.

**Figure 1:**
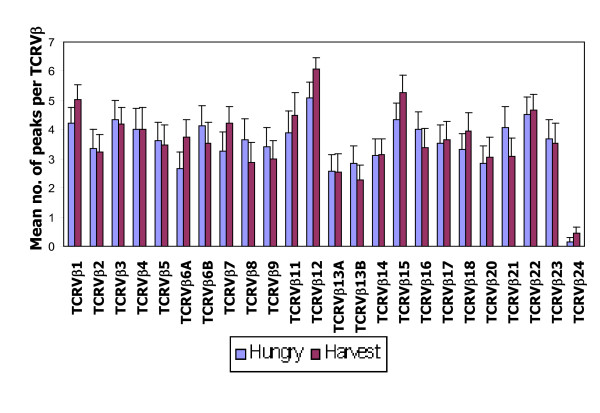
**Season of birth patterns, for mean number of TCRVβ peaks in the 24 TCRVβ types**. The mean numbers of peaks per TCRVβ type (a measure of T cell clonal diversity) which is calculated as the average of the sum of the peaks in each of the 24 TCRVβ types per season are shown for the hungry/high infection (blue) and harvest/low infection (maroon) seasons. The error bars represent 1SE from the mean. The Fig shows that the average numbers of peaks for virtually all 24 TCRVβ types are much lower than the 8-10 peaks seen in healthy adults. There were 26 subjects for each season of birth. Except for TCRVβ24 for which the hungry/high infection season average was significantly lower than for the harvest/low infection season average (p < 0.03), there were no significant season of birth differences in the overall number of TCRVβ peaks indicating the absence of seasonal effects on overall CD8^+ ^T cell clonal diversity.

The number of CDR3 peaks provides a measure of T cell clonal diversity. Peaks representing genuine T cell clones were defined as those with fluorescence intensities above 500, an arbitrary 'cut off' which excluded background 'noise' fluorescence. Figure 1 shows that the average number of peaks for virtually all 24 TCRVβ types are much lower (<6 per TCRVβ) than the 8-10 peaks seen in healthy adults [32], reflecting oligoclonal expansions characterizing repertoire skewing. The results also revealed that there were no season of birth differences (p values on Table 3) in the total number of peaks generated by individual TCRVβ types except for TCRVβ24 for which, those born in the hungry/high infection season had lower peak numbers (p < 0.03). TCRVβ12 had the highest mean number of peaks in both seasons-of-birth and TCRVβ24 generated the lowest mean (Figure 1). All but 8 subjects (1 hungry/high infection and 7 harvest/low infection season born) failed to produce any peaks for TCRVβ24.

**Table 3:** TCRVβ usage, as fluorescence intensity-calculation of individual TCRVβ types of CD8^+ ^subset in the population and by season of birth in 18-23 years old men.

TCRVβ Type	Mean fluorescence intensityx10^4^/subject			*P value
		
	All (52)	Harvest season (n = 26)	Hungry season (n = 26)	
**1**	6.4	6.8	6.0	0.59

**2**	2.8	3.1	2.6	0.97

**3**	6.3	6.6	6.0	0.88

**4**	6.4	7.5	5.2	0.41

**5**	5.2	5.0	5.2	0.88

**6A**	3.8	4.1	3.5	0.30

**6B**	4.7	4.5	4.9	0.68

**7**	4.1	4.8	3.5	0.27

**8**	5.1	4.8	5.9	0.35

**9**	4.4	4.0	4.8	0.93

**11**	6.4	7.3	5.5	0.22

**12**	9.6	11.6	7.6	0.04

**13A**	2.4	2.5	2.4	0.98

**13B**	3.5	3.8	3.3	1.00

**14**	4.2	4.2	4.3	0.94

**15**	5.9	6.2	5.5	0.65

**16**	5.6	4.9	6.2	0.27

**17**	5.2	5.6	4.8	0.54

**18**	3.7	3.6	3.8	0.82

**20**	3.6	3.5	3.7	0.75

**21**	5.6	5.2	6.0	0.36

**22**	6.0	6.2	5.7	0.60

**23**	4.9	5.0	4.9	0.87

**24**	0.3	0.1	0.4	0.03

*P value are for differences between TCRVβ usage by season of birth, with the number of subjects (n) shown.

**Figure 2:**
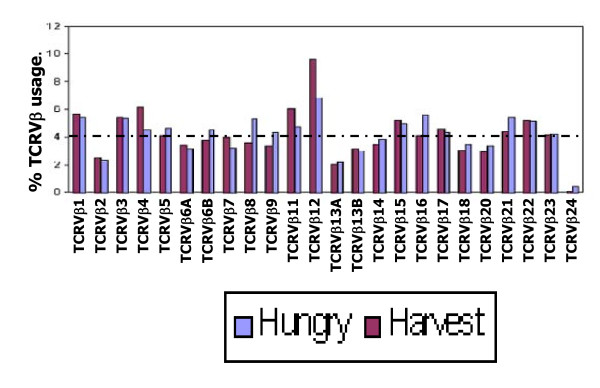
**Relative CD8^+ ^TCRVβ usage by season of birth, in the CD8^+ ^T cell subset**. The relative usage within the hungry/high infection or harvest/low infection season for each of the 24 TCRVβ types is determined by expressing the sum of the fluorescence intensities of each TCRVβ type as a percentage of the total of all peak fluorescence intensities in the population. The percentage CD8^+ ^TCRVβ usage for those born in hungry/high infection season (blue bars) and harvest/low infection season (maroon bars) are shown. While the usage of most TCRVβ types did not exceed 5%, TCRVβ12 usage was consistently higher than all TCRVβ types for both seasons of birth. The near extinction of TCRVβ24 is manifested in the near zero usage observed for both seasons of birth. There were 26 subjects each born in the hungry/high infection or harvest/low infection seasons. If each TCRVβ type was used at same frequency, usage would be expected at a frequency of approximately 4% (1/24) [dotted horizontal line], therefore there is an overall high usage; with most (16/24) showing ≥4% usage. TCRVβ12 'publicly' expressed perhaps reflecting association with the endemicity of hepatitis B virus; TCRVβ12 being specific for HBV core antigen. Significant season of birth differences were seen for TCRVβ12 and 24; p = 0.04 and p < 0.03 respectively.

While peak numbers define clonal diversity, total fluorescence intensity of a TCRVβ type

determines clonal abundance. Expressing the sum of the fluorescences generated by each of the 24 CD8^+ ^TCRVβ CDR3 spectra-types as a percentage of the sum-total of fluorescences generated by all TCRVβ spectra types, provides the percentage TCRVβ type usage. The results showed an average usage of less than 5% for the majority of TCRVβ types. However, the usage of CD8^+ ^TCRVβ12 for those born in the harvest/low infection season was approximately twice the population mean (Figure 2). Further statistical analysis confirmed that the usage of CD8^+^TCRVβ12 for those born during the hungry/high infection season was significantly lower than for those born during the harvest/low infection season at 7.76 versus 10.24 × 10^4 ^mean fluorescence intensity respectively (p = 0.04; Table 3).

The analyses also revealed, that while CD8^+^TCRVβ12 was universally (100%) expressed, CD8^+ ^TCRVβ24 was virtually deleted in the study population. Seven out of the 26 subjects (26.9%) born in the harvest/low infection season showed modest expression of the CD8^+ ^TCRVβ24 compared to only 1 of the 26 (3.8%) for those born during the hungry/high infection season, representing 73% and 96% deletion of TCRVβ24 respectively. TCRVβ22 was also expressed in all but 1 subject who was born in the hungry/high infection season. All those born in the harvest/low infection season showed good expression of TCRVβ22.

Peculiar expression patterns were also noted for TCRVβ5, 8 and 20, with deletions observed in 21/52 (40%), 22/52 (42%) and 24/52 (46%) respectively. However there were, no season of birth differences in the expression of these TCRVβ types.

### Season of birth differences evident in the CD8^+^, but not CD4^+ ^telomere lengths

Relative telomere length analyses were performed on 58/60 CD4^+ ^and 60/60 CD8^+ ^samples. The results revealed that average telomere length for the CD8^+ ^T cell subset was 3 fold shorter than for the CD4^+ ^subset with GM Ct values at 0.06 and 0.02/100 cells for the CD8^+ ^and CD4^+ ^subset respectively, p < 0.001. The average telomere length for the CD8^+ ^subset of those born in the hungry/high infection season was marginally lower than for those born in the harvest/low infection season, with GM Ct values 0.02, versus 0.03, per 100 cells for the harvest/low infection hungry/high infection seasons respectively, p = 0.05 (Table 4).

**Table 4:** CD4^+ ^and CD8^+ ^relative telomere length, in the population and by season of birth in 18-23 years old men.

	GM Ct/100 cells				
	**CD4^+ ^** **(n)**	**95% CI**	**CD8^+ ^** **(n)**	**95% CI**	*P value

**All Subjects**	0.02 (58)	0.014-0.039	0.06 (60)	0.036-0.112	<0.05
**Harvest**	0.02 (30)	0.009-0.040	0.01 (30)	0.007-0.027	0.5†
**Hungry**	0.03 (28)	0.010-0.050	0.04 (30)	0.018-0.069	0.62†
**P value**	0.49		0.05		

GM cross over time (C_t_) which is inversely proportional to telomere length, number of subjects (n), 95% CI and p values are shown.

## Discussion

We previously showed associations between season-of-birth, thymic size and functional changes during early infancy, with those born during the harvest/low infection season having larger thymi and enhanced T cell production[3]. To test the hypothesis that these early life events are amplified in adults to reflect the season of birth effects on the reported adult mortality,[2] we first assumed that as infants, the young adults studied here, were exposed to some of the same seasonal pressures which existed previously, although current improvements in the socioeconomic conditions of today are consistent with milder environmental pressures. The improved socioeconomic conditions coupled with the repeated exposure of environmental stressors across the seasons has the potential to obscure previously reported season of birth differences[2]. Unlike the earlier findings in the babies, [3] we did not observe season-of-birth associations with thymic output in the young adults. In addition to the overall improvements in the socioeconomic conditions enjoyed today, it is also possible that the cumulative impact of chronic nutritional deprivation and repeated infections over the years leading to adolescence obscured season-of-birth differences on thymic output which may have pre-existed at infancy. The hungry/high infection season is associated with greater mortality rates; [1] therefore it is also possible that the worst affected individuals died before reaching adolescence which may be a source of bias. It support of our data, the lower TREC concentrations in the young adults compared to the reports in the babies, [3] are consistent with reduced thymic output in advancing age[34].

Despite the lack of season of birth effects on thymic output, lower CD4^+ ^as well as CD3^+ ^counts were associated with hungry/high infection season births. Considering the central role of CD4^+ ^cells in the immune system, [35-38] this implies worse immunity for adults born in the hungry/high infection season. When season of collection was controlled for, (i.e. sampling done in a different season to the subject's birth) this did not significantly alter the findings, although sample sizes were substantially reduced. Nonetheless, 67.9% (38/56) for the CD4^+ ^T cell subset were collected during the hungry/high infection season months of July to December, therefore it is possible that the raised CD4^+ ^and CD3^+ ^counts for those born in the harvest/low infection season (but had some of their samples collected in the hungry/high infection season) were partly influenced by more peripheral T cell proliferation. Antigenic load which drives T cell proliferation is higher in the hungry/high infection season when infectious burden is heavier;[39] and malaria which peaks here during the hungry/high infection season, is likely to impose further immune pressure accompanied by changes in T cell counts as reported in human studies[40,41]. Further studies specifically testing the season of collection are needed to verify this.

The lack of season of birth effects on thymic output may reflect its higher tolerance threshold, than the CD4^+ ^and CD3^+ ^subsets, which may be more sensitive to pressures from environmental elements. In support of this, dietary zinc was associated with and differential rises of up to 24% and 64% CD4^+ ^and CD3^+ ^lymphocyte counts respectively[42].

Similarly to the CD4^+ ^and CD3^+ ^counts, the season of birth effects on specific TCRVβ types suggested differential sensitivities to elements of the environment. The reduced usage of TCRVβ12 for those born in the hungry/high infection season (most of whose samples were also collected in the hungry/high infection season) may be related to the prevailing infections. HBV infection is endemic in this community with 10-15% of adult male deaths due to hepatocellular carcinoma which is associated with HBV infection[43] and it has been shown that TCRVβ12 is specific for hepatitis B virus (HBV) core antigen[44]. Those born in the hungry/high infection season may be less able to control HBV (particularly during the hungry/high infection season), possibly leading to increased liver damage[45]. The season of birth differences observed for the TCRVβ24 which is deleted in all but 8 of the 52 subjects all of whom only lowly expressed TCRVβ24, suggested that TCRVβ24 may not be of significant value for immune protection in either season. It is possible that a 'chance' Type 1 statistical error arising from multiple testing of repeated variables may account for the season of birth difference seen, and that a bigger sample size may produce significant differences in other TCRVβ types.

Repertoire skewing is consistent with accelerated proliferation and the potential to drive telomere erosion, therefore the shorter mean telomere length for those born in the hungry/high infection season suggested that their CD8^+ ^T cells were under proliferative pressure and at a higher risk of replicative senescence. Telomere shortening is accelerated in arterial tissue exposed to oxidative stress factors including reactive oxygen species (ROS)[46]. The endemicity of infections in this community may be expected to generate ROS to contribute to telomere shortening especially for those born in the hungry/high infection season.

To optimize the interpretation of our TREC findings at the population level, results from other study populations were used for comparison. The TREC assay which is now widely used as a marker of thymic output lacks a 'gold' standard; thus limiting the number of studies with which to compare our data. However, our results suggested that average TREC concentrations in the subjects studied may be substantially lower than those of adults elsewhere, [47,48] implying diminished thymic output and immune capacity in this population. Persistent infectious burden rather than low thymic output may also be responsible for the lower TRECs; since elevated cell proliferation from antigenic exposure is known to dilute TREC concentrations[49]. Our findings, at the population level, that the T cell subsets are comparable to those of healthy individuals from the sub region, [50-53] imply that poor T cell immunity may be common here. The lack of observable differences arising from the further analyses by birth weight category (higher/lower than the population median) is consistent with the overall findings but may have been confounded by the resultant reductions in numbers.

Our analyses of the T cell repertoire was meant to give an in depth evaluation of T cell immune status beyond thymic output and T cell numbers, and the extensive CD8^+ ^TCRVβ repertoire distortions in the population indicated more severe immune challenges than was evident from the thymic output and T cell counts. Only 2 (TCRVβ12 and 15) out of 24 TCRVβ types showed an average spectra-type peak number ≥5 across all donors; compared to reports of >8 peaks in healthy individuals[54,55]. We speculate that repertoire skewing in this population was driven by environmental stressors including the repeated persistent antigenic exposure annually and across the seasons due to the endemicity of infections [56-58] including CMV, which is associated with virus specific CD8^+ ^T cell types and other risk factors [59,60]. We argue that the chronic nature of the assault on the immune system of both groups may be the reason for the general distortion of the TCRVß repertoire. Significantly, the timing of exposure to environmental stressors may be more critical, the closer to the time of birth it occurs, as the thymus experiences its greatest and only growth phase in the first year of life, a period of maximum vulnerability; with the potential to generate ever-lasting impact on the thymus and the T cells it generates. Consequently the thymi of those born in the hungry/high infection season may never be adequately compensated to cope with later life demands. Conversely, thymi of those born in the harvest/low infection in a more enabling environment for development, may be endowed with a more resilient initial thymic capacity. The immune insufficiency implied by the apparent oligoclonal repertoire distortions is consistent both with the lower thymic output compared to others;[47,48] and supported by the association of a polyclonal repertoire with a lack of antigen exposure,[61] favourable immunity being associated with good thymic output and a broad repertoire[62].

Chronic HBV infection is also endemic in this community,[63] and the publicly expressed TCRVβ12 being specific to the HLA-A2 restricted hepatitis B virus (HBV) core antigen[44] supports a role for HBV in the marked global repertoire skewing seen. The near extinction of TCRVβ24 in the population, which has also been reported in other settings, where TCRVβ24 became notably expanded when stimulated by specific antigen,[61] suggested that the near zero expression in our study was probably not due to lack of capacity for the TCRVβ24 clone to expand. This implies that TCRVβ24 offers little, if any, advantages in this community. As clonal expansion and cell division are accompanied by telomere erosion,[64,65] the shorter telomere of the CD8^+ ^compared to the CD4^+ ^subset, supports reports that the CD8^+ ^subset undergoes faster clonal expansion[66]. Indeed shorter mean telomere length has previously been reported for the CD8^+ ^compared to the CD4^+ ^subset in healthy humans[67] and with increasing T cell turnover[65]. A naïve T cell is estimated to go through at least 14 cell divisions during an immune response,[68,69] therefore the repeated infections might be expected to drive telomere shortening although human studies with which to compare our data were lacking.

## Conclusions

Taken together, our data showed no definitive link between adult thymic function and early life effects. Despite this, season of birth differences in the CD4^+ ^and CD3^+ ^counts as well as CD8^+ ^telomeres, suggest that aspects of adult T cell immunity may be under the influence of early life stressors. We also argue that, repeated annual cycles of nutritional deprivation and infectious burden may drive overall CD8^+ ^TCRVβ repertoire skewing possibly related to risk factors including CMV and HBV infections. Put together, we propose the environmental pressures possibly of nutritional origin, predispose this population to infections arising from the resultant challenges to the immune system.

## Competing interests

The authors declare that they have no competing interests.

## Authors' contributions

PTN**, **AMP and RA conceptualized, designed the study and participated in drafting the manuscript. PTN did the laboratory work including all the molecular analyses. **JS **participated in the field work; GM and SEM participated in drafting the manuscript. All authors read and approved the final manuscript.
